# Utilization of Simulation Techniques to Enhance Quality Improvement Processes in the Emergency Department

**DOI:** 10.7759/cureus.7009

**Published:** 2020-02-16

**Authors:** Geoffrey B Comp, Benjamin V Silver, John Elliott, Andrew Kalnow

**Affiliations:** 1 Emergency Medicine, Creighton University School of Medicine/Maricopa Medical Center (Phoenix), Phoenix, USA; 2 Emergency Medicine, OhioHealth Doctors Hospital, Columbus, USA; 3 Medical Education, OhioHealth Riverside Methodist Hospital, Columbus, USA

**Keywords:** emergency medicine, medical simulation, quality improvement

## Abstract

Introduction

Quality improvement projects can help improve clinical practice in an emergency department (ED). However, it is difficult to measure outcomes in rare clinical conditions. We used a simulation program to evaluate a new protocol and workflow in the emergency blood transfusion process as well as provide additional trauma training. To determine if implementing a trauma simulation would help improve the self-reported understanding of the emergency blood transfusion process by both the ED and laboratory staff.

Methods

Emergency medicine residents and nursing staff participated in a high-fidelity trauma simulation. ED nursing and hospital laboratory staff used the simulation to test a new process for notification and transport of blood within the hospital. All of the participants were provided a four-item Likert scale questionnaire immediately after the training to evaluate their understanding of the ED blood process.

Results

There was a significant improvement in overall scores based on paired t-tests in the full group (pre 15.0 versus post 17.6, p = 0.0005) and ED group (pre 14.7 versus post 17.8, p = 0.0007) but not in the lab group (pre 15.8 versus post 17.2, p = 0.296).

Conclusion

Simulation appears to be helpful to evaluate and implement a new ED protocol or workflow.

## Introduction

The emergency department (ED) represents a rich environment for quality improvement (QI) initiatives due to the high-volume, high-acuity nature of emergency medicine practice, and provides many potential areas for improvement in protocols and patient safety. Thus, the ED has become an important arena for testing and implementing healthcare QI projects [1]. However, it can be difficult to measure process improvement outcomes if an examined process involves a rarely occurring event.

The use of high-fidelity medical simulation has demonstrated effectiveness in the education of medical residents. Simulation allows the creation of cases that test specific skills or thought processes by guiding the learner through critical actions and responding to participant actions [2]. Confidence, problem-solving, and uncommon procedures can be tested and rehearsed in a simulated environment [3]. Similarly, specific protocols and processes can be tested with simulation [4]. 

In surgery, simulation has been used to reproduce adverse events [2]. When evaluating malpractice cases that had previously undergone root cause analysis, simulation identified system errors that had previously been missed. Root cause analysis is retrospective, relying on participant memory and medical documentation, while simulation allows a prospective, dynamic form of error analysis which can be recorded and analyzed multiple times. By interviewing participants, simulation allows evaluation of naturalistic decision making, by identifying which cues individuals use during problem solving. In addition, it allows training for high-risk, low-volume events such as malignant hyperthermia [3]. Such training is crucial for maintaining coordination between members of multidisciplinary teams during high-acuity events, and is most effective when simulations involve members from all relevant medical disciplines.

In an ED context, in situ simulation has been used successfully to orient ED staff to new facilities prior to opening and assess an active ED for latent safety threats (LSTs) during regular operations [4-6]. In addition, simulation represents a medium for original research into team dynamics and decision making [7]. These simulations not only detect LSTs at a higher rate than when performed in a simulation lab, but they also offer an opportunity to continually practice both technical and non-technical (communication) skills amongst ED team members without endangering patients. In fact, due to the success of one of these studies, an ED made in situ simulation a regular part of their monthly schedule and directly linked it to a QI committee [8]. 

We used the simulation program to examine a new QI project trialing the impact of a new workflow in the hospital-wide emergency blood transfusion protocol. It also provided benefit to the residents and time to rehearse trauma management and care.

## Materials and methods

This was a prospective one-sample post-test educational evaluation. The simulations took place across two months in the ED of a community-based hospital. In order to assess the status of our critical care/trauma treatment process, a simulation series was developed to be implemented in an active ED. We utilized a resuscitation room, ED staff, and EM residents to perform the in situ simulated patient scenario. High-fidelity patient simulators and task trainers were placed in the ED resuscitation room to be used during the simulation. Care was taken to target rapid treatment of an unstable trauma patient including activation of the hospitals emergent trauma blood process which was being developed with ED administrative and clinical staff.

The specific case for this session was designed to trigger our highest trauma level activation which requires uncross-matched blood be brought to bedside. We observed how the case was handled by the medical providers and the blood bank through the new ED process. We planned to assess areas of breakdown in the process to further streamline and improve treatment in a time critical patient encounter. 

It was hypothesized that after the simulation, the self-reported understanding of the emergency blood transfusion process would improve. Nursing, physician, and additional medical personnel who participated in the in situ simulation, as well as laboratory and blood bank personnel, were then surveyed to determine their understanding of the ED blood policy. 

The survey consisted of four questions for the participant to self-report their confidence and understanding of the emergency blood protocol. Participants were instructed to assess the usefulness of the simulation training regarding the new blood process policy using a five-point modified Likert scale with 5 for “strongly agree” to 1 for “strongly disagree.” There was also a single write in line asking for any additional comments. Participants evaluated these items at completion of the course and retrospectively evaluated the same items before the course. The items were summed to yield a total score with possible points ranging from 4 to 20. Statistical analyses were conducted using paired t-tests in Excel, which reports means and p-values.

## Results

Data were gathered on 17 participants: 12 in the ED group and 5 in the lab group. Descriptive information from the post-test/retrospective pre-test Likert scale questions are shown in Table 1, which shows a positive shift in agreement on all four questions. 

**Table 1:** Retrospective pre-test post-test course evaluation (n = 17)

Before the course, % (n)					Assessment question	At the end of the course, % (n)				
Strongly disagree 1	Disagree 2	Neutral 3	Agree 4	Strongly agree 5		Strongly disagree 1	Disagree 2	Neutral 3	Agree 4	Strongly agree 5
0 (0)	5.9 (1)	41.2 (7)	29.4 (5)	23.5 (4)	I feel confident in how to activate the process for the emergency blood protocol at Doctors Hospital	5.9 (1)	0 (0)	0 (0)	41.2 (7)	52.9 (9)
0 (0)	5.9 (1)	29.4 (5)	29.4 (5)	35.3 (6)	I know the specific situation to activate the process for the emergency blood protocol at Doctors Hospital	0 (0)	5.9 (1)	0 (0)	35.3 (6)	58.8 (10)
0 (0)	29.4 (5)	17.6 (3)	17.6 (3)	35.3 (6)	I know how to identify the “runner” on the daily assignment sheet	0 (0)	5.9 (1)	0 (0)	17.6 (3)	47.1 (8)
0 (0)	23.5 (4)	11.8 (2)	29.4 (5)	35.3 (6)	I understand the process for getting emergency blood from the blood bank to the emergency department	0 (0)	0 (0)	5.9 (1)	11.8 (2)	82.4 (14)

Due to the small sample size, we are underpowered for a statistical comparison of all questions. Thus, scores from these questions were summed and compared. There was a significant improvement in overall scores based on paired t-tests in the full group (pre 15.0 versus post 17.6, p = 0.0005) and ED group (pre 14.7 versus post 17.8, p = 0.0007) but not in the lab group (pre 15.8 versus post 17.2, p = 0.296). 

Table 2 describes a post-evaluation assessment question from 16 of the participants that either agreed or strongly agreed that “The simulation component for obtaining emergency blood helped me familiarize myself with the process." Open-ended comments were also very supportive of the training, as shown in Table 3. 

**Table 2:** Participant familiarity with the trauma process

Assessment question		At the end of the course, % (n)				
		Strongly disagree 1	Disagree 2	Neutral 3	Agree 4	Strongly agree 5
The simulation component for obtaining emergency blood helped me familiarize myself with the process (n = 16)	0 (0)		0 (0)	0 (0)	12.5 (2)	87.5 (14)

**Table 3:** Additional recommendations for future training events

Do you have any other comments?	Response
	“Should run sim with problems to see how people react and respond.”
	“Now having the blood @ the bedside attaching the rapid infuser is beneficial.”
	“Great!”
	“We need to keep doing sims!”

## Discussion

The use of simulation for medical education has been readily utilized especially for the evaluation of medical techniques and procedures as well as evaluating and teaching effective communication procedures and interpersonal interactions. However, there is limited data on the use of simulation to help introduce ED staff and stakeholders to a new ED workflow procedure. This initiative was unique in that multiple departments within the hospital participated in the simulation to test their own specific roles in the process as well as the overall efficacy.

The simulations helped identify potential areas for improvement and practice with many different members of the ED staff. We observed major areas for improvement include autoinitiation of the process for any category one trauma patient, generating a “Jane/John Doe” patient identification number, and tasking a specific person to act as a courier to retrieve the blood from the lab and blood bank. Recorded data points included time of prehospital category one activation, notification of blood bank by ED charge nurse, runner arriving to blood bank, time of release of blood from the blood bank, and blood arrival to resuscitation room. As there was not a statistically significant improvement in understanding of the process with the lab personnel, we used this as an opportunity for additional education with the laboratory and blood bank staffing. These will be evaluated for future projects to optimize timing. 

While the data suggest this method may be a way to test a new workflow pattern and provide staff training, there are some limitations to this evaluation. First, as this was an observational study with pre and post self-assessment, there is a potential for self-reporting bias. Additionally, the study sample size was relatively small, and can be expanded in the future for additional opportunities. As there was no standardization of the participants including level of medical training in the simulation, there is also a potential to expand the program to allow for additional participation.

## Conclusions

This model of using simulation to assist with training of more than medical skill and knowledge can be expanded to test and evaluate different policies and procedures. There is potential to use simulation to help test new policies, assist in new provider orientation, as well as demonstrating potential areas to assist with problem solving and collaboration. 
